# Evaluation of the Dentinal Shear Bond Strength and Resin Interface in Primary Molars after Pre-Treatment with Various Dentin Bio-Modifiers: An In Vitro Study

**DOI:** 10.3390/jfb15020041

**Published:** 2024-02-11

**Authors:** Saima Sultan, Seema Chaudhary, T. R. Chaitra, Naveen Manuja, Sinha Ashish Amit, Mamata Iranna Hebbal, Alhanoof Aldegheishem, Selma A. Saadaldin, Elzahraa Eldwakhly, Amal Ali, Mai Soliman

**Affiliations:** 1 Department of Pedodontics and Preventive Dentistry, Kothiwal Dental College and Research Centre, Moradabad 244001, India; saimasultan8076@gmail.com (S.S.); chaudharyseema2@gmail.com (S.C.); chaitumbd@gmail.com (T.R.C.); naveenmanuja@gmail.com (N.M.); sinhashish@gmail.com (S.A.A.); 2 Department of Preventive Dental Sciences, College of Dentistry, Princess Nourah bint Abdulrahman University, P.O. Box 84428, Riyadh 11671, Saudi Arabia; mihebbal@pnu.edu.sa; 3 Department of Clinical Dental Sciences, College of Dentistry, Princess Nourah bint Abdulrahman University, P.O. Box 84428, Riyadh 11671, Saudi Arabia; asaldegheishem@pnu.edu.sa (A.A.);; 4 Prosthodontics Division, Schulich School of Medicine and Dentistry, Western University, London, ON N6A 3K7, Canada; ssaadal@uwo.ca; 5 Faculty of Dentistry, Alexandria University, Alexandria 21526, Egypt

**Keywords:** dentin bio modifiers, resin interface, shear bond strength, SEM

## Abstract

Dentine adhesives have demonstrated great success with permanent teeth. Though the results in primary teeth are not well documented, some studies have demonstrated lower values of bond strength in primary teeth than those found in permanent teeth. The aim of this study was to compare and evaluate the effect of grape seed extract (6.5%) (Herbal Bio Solutions, Delhi, India), glutaraldehyde (5%) (Loba Chemie PVT. LTD., Mumbai), hesperidin (0.5%) (Herbal Bio Solutions, Delhi, India), and casein phosphopeptide-amorphous calcium phosphate (tooth mousse) (GC Corporation, Alsip, IL, USA) on the shear bond strength of dentine of primary teeth and to evaluate the resin tags at the resin tooth interface. Seventy-five caries-free human primary molars were collected, and their occlusal surfaces were ground flat. Dentin surfaces were etched using phosphoric acid. Then teeth were randomly assigned in sequential order to five groups according to the dentinal treatment method: Group I (Control group) (no treatment), Group II (5% glutaraldehyde), Group III (6.5% grape seed extract), Group IV (0.5% hesperidin), and Group V (CPP-ACP). Ten teeth from each group were assigned for Shear Bond Strength and five for SEM analysis. ANOVA and a post hoc least significant difference test (*p* < 0.05) were used for statistical analysis of the collected data. The grape seed extract group showed significantly increased shear bond strength than the control group (*p* < 0.05), and the mean length of resin tags in different dentine bio modifiers groups was also statistically significant (*p* < 0.05). The use of dentin bio modifiers such as 5% glutaraldehyde, 6.5% grape seed extract, 0.5% hesperidin, and CPP-ACP in the bonding process for primary teeth did not improve the dentinal bond strength.

## 1. Introduction

One of the most effective ways of improving the mechanical bonding and marginal seal of the restoration to dentin is primarily through proper acid-etching, which provides an excellent mechanism for stable bonding and long-lasting resin restorations [1]. Dentin bonding relies on hybrid layer formation, a bio-composite that includes dentin collagen and polymerized resin adhesive and is considered a resin-dentin interdiffusion zone [2]. Dentinal collagen can be strengthened by native crosslink formation, which provides fibrillar resistance against enzymatic degradation, and by improving dentinal mechanical properties, including tensile strength [3].

Although research in this area has been in progress since the 1950s, dentin still poses greater obstacles to adhesive bonding compared to enamel, especially in primary teeth, owing to the chemical, physiological, and micromorphological differences between dentine of primary teeth and dentine of permanent teeth [4]. Preserving the collage matrix integrity is the main reason to improve dentin bonding stability [2] and to infiltrate the adhesive material into the dentin collagen matrix, which is usually exposed after etching [5]. In addition to the challenges of bonding dentin in general, the unique nature of the dentine of primary teeth makes it more resistant to ideal bonding due to the previously mentioned histological and morphological differences. Additionally, primary teeth are more sensitive to acidic treatment due to their reduced mineral content [6]. Also, the dentin of the primary teeth has a wider tubule diameter, leading to a reduction in the intratubular dentin area available for bonding [7]. Other factors that limit bonding in primary teeth are the variations in the intrinsic humidity and permeability of the primary dentine and the contradictory data regarding hybrid layer formation [8]. Accordingly, different studies have reported lower bond strength values in primary teeth dentine compared to permanent teeth dentine [6,9,10].

Despite the deterioration associated with the degradation of the dentin-adhesive interface over time, research continues to improve the integrity of this interface. One such researched technique is using extrinsic collagen cross-linking agents to improve the intrinsic properties and modify the dentin by promoting resistance to enzyme degradation and inhibiting the activity of extracellular matrix metalloproteinases and cysteine cathepsins that could hydrolyze the organic matrix of the conditioned dentin, triggering hybrid degradation [5]. Extrinsic collagen cross-linking agents could be either synthetic agents, nature-derived agents, or physical means to interact effectively with type I collagen [11].

Various studies have investigated the effect of treating dentin with natural or synthetic crosslinking agents as dentin bio modifiers [12,13]. The mechanism of action of the cross-linking agents is to develop mechanically stable collagen scaffolds and to improve dentine penetrability [14]. Nature-derived grape seed extract composed mainly of proanthocyanins proved to modify the physical properties of dentin by inducing cross-linking with collagen, increasing the stiffness of demineralized dentin, and inhibiting metallo-proteinase matrix formation [15,16]. Synthetic glutaraldehyde is a cross-linking and disinfecting agent that improves dentin’s mechanical properties by inducing cross-linking formation through reaction with ε-amino groups [17]. Similarly, hesperidin extracted from citrus fruits has been shown to inhibit the degradation of collagen [18]. Another novel approach is dentine pre-treatment using a remineralization concept based on casein phosphopeptide-amorphous calcium phosphate (CPP-ACP), which proved to increase the bond strength in tridimensionality. Borges et al., reported a higher push-out bond strength of dimethacrylate and silorane-based restorative systems after careful dentin pre-treatment with a paste containing CPP-ACP (MI Paste). Recent studies claimed that dentin samples treated with remineralization paste had statistically higher bond strength means than untreated samples [19]. Nevertheless, few studies have evaluated the efficacy of dentine bio modifiers on adhesive bond strength in primary teeth.

Consequently, the primary aim of the present study was to examine the effects of various dentin bio-modifiers on the shear bond strength of primary teeth and their influence on the resin-dentin interface. We hypothesize that the induction of dentine bio modifiers improves dentin collagen stability and thus increases bond strength in primary teeth. 

## 2. Materials and Method

The study was carried out in accordance with the Code of Ethics of the World Medical Association (Declaration of Helsinki). Ethical approval was obtained from the Ethical Committee on Human/Animal Subjects Research at Kothiwal Dental College and Research Centre (Rf No. KDCRC/ETH/PEDO/2013/03-01, 9 April 2013).

**Figure 1:**
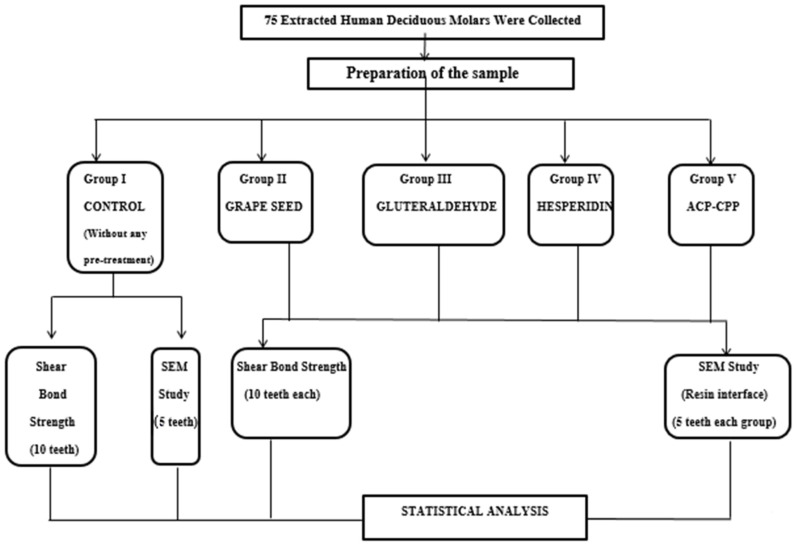
Schematic illustration of the study design.

A total of 75 primary, non-carious molars were used in the study. The collected teeth were caries-free, non-hypoplastic, without enamel cracks, and had pre-shedding mobility. All the teeth were cleaned with prophylactic instruments and stored in normal saline until use. The teeth were randomly divided into five groups: one control group and four experimental groups. Fifteen teeth in each group were found to be sufficient based on the previous studies [20,21,22], thus comprising a sample size of 75 (Figure 1).

### Experimental Groups

All teeth were randomly divided according to the dentin treatment into five groups as follows: control group (with no treatment done), 5% glutaraldehyde (Loba Chemie Pvt Ltd., Mumbai, India), 6.5% grape seed extract (Herbal Bio Solutions, Delhi, India), 0.5% hesperidin (Herbal Bio Solutions, Delhi, India), and CPP-ACP (GC Corporation, Alsip, IL, USA).

## 3. Solution Preparation:

### 3.1. Grape Seed Extract Treatment Group (GSE)

#### 3.1.1. Preparation of 6.5% Grape Seed Extract Solution

Approximately 6.5% grape seed solution was made by the saturation method [23]. Commercially available 95% grape seed powder was added to 100 mL of distilled water until saturation was achieved. The solution was heated continuously for 1 hour to achieve a 100% grape seed solution. To make 6.5% of GSE solution, 93.5 mL of distilled water (DW) was added to 6.5 mL of GSE solution. A total of 93.5 mL of DW + 6.5 mL of 100% GSE solution = 6.5% of GSE solution.3.2. Glutaraldehyde Treatment Group (GD)

#### 3.1.2. Preparation of a 5% Glutaraldehyde Solution

A 5% solution of glutaraldehyde was made by the dilution method. A commercially available 25% glutaraldehyde solution of 100 mL was diluted by adding 500 mL of distilled water (solvent). Approximately 25% GD of 100 mL + 500 mL of distilled water = 5% GD solution.

### 3.2. Hesperidin Treatment Group (HPN)

#### Preparation of a 0.5% Hesperidin Solution

Approximately 0.5% of Hesperidin was also prepared by the saturation method. Commercially available 10% Hesperidin (HPN) powder was added to 100 mL of distilled water until saturation was achieved. The solution was heated continuously for 1 h. Thus, a 100% HPN solution was achieved. Now, 99.5 mL of distilled water was added to 0.5 mL of 100% HPN solution to achieve the final solution of 0.5% HPN.

The exposed dentin surface was treated with a bio modifiers solution using an applicator brush for 1 min, then rinsed with water and blot dried, followed by the bonding/composite buildup procedure.

### 3.3. Casein Phosphopeptide-Amorphous Calcium Phosphate Treatment Group (CPP-ACP)

The exposed dentin surface was treated with CPP-ACP, Tooth Mousse, using an applicator brush for 1 min, rinsed with water, and blot dried, followed by the bonding/buildup procedure.

In the control group, no pre-treatment was conducted. While for each experimental group, 15 teeth were pre-treated with the respective dentin bio modifier, ten teeth were tested for shear bond strength, and five for Scanning electron microscopy (SEM, Kyoto, Japan) analysis.

## 4. Laboratory Analysis

### 4.1. Sample Preparation for Shear Bond Strength

The selected teeth were embedded perpendicular to their long axis into the acrylic resin mold with occlusal surfaces facing upwards. The occlusal surfaces were ground flat using a double diamond disk with a diameter of 22 mm under running water to grind the enamel completely and expose dentin. The flat dentin surfaces were acid-etched for 15 s with 37% phosphoric acid, and teeth were randomly divided according to the pre-treatment and bonding system used.

In Group I (the control group), no pre-treatment was conducted on the exposed dentin surface. Two successive coats of the adhesive Adper Single Bond 2 (3EM ESPE, 3M, St. Paul, MN, USA) with compositions such as BisGMA, HEMA, dimethacrylates, ethanol, water, a novel photo initiator system, and a methacrylate functional copolymer of polyacrylic and polyitaconic acids, were applied to the prepared dentin surface of the specimens according to the manufacturer’s instructions, air dried, and light cured using the QTH Light Curing Unit (QTH Spectrum, Dentsply, Charlotte, NC, USA) for 20 s. 

Composite build-up (Esthet X HD, Dentsply with composition as Bis-GMA adduct, a Bis-EMA adduct, and triethylene glycol dimethacrylate, Camphorquinone (CQ), Photoinitiator, Stabilizer, Pigments) was conducted by placing two increments of 2 mm thickness and diameter same as that of the occlusal surface of the tooth using stainless steel composite applicators; each increment was light cured for 20 s using QTH light curing unit (QTH Spectrum, Densply). 

In the experimental groups, the exposed dentin surfaces were treated with 6.5% grape seed extract solution, 5% glutaraldehyde solution, 0.5% hesperidin solution, and CPP-ACP (Tooth Mousse), respectively, using an applicator brush for 1 min and then rinsed with water and blot dried, followed by the bonding/composite build-up procedure as described above (2 increments of 2 mm thickness). The specimens were stored in distilled water until subjected to the testing procedures. 

### 4.2. Shear Bond Strength Test (SBS)

All the prepared samples were subjected to a shear bond strength test after one week of storage in distilled water using a universal testing machine (Instron machine, ADMET, Enkai Enterprises, New Delhi, India) at a cross-head speed of 0.5 mm/min using a straight knife-edge chisel applied at the tooth restoration interface until fracture occurred, using a 100 N load cell. The load was applied until a restoration failure occurred. The shear bond strength was calculated in megapascals (Mpa). The results were tabulated and statistically analyzed. The shear bond strength data were analyzed by one-way ANOVA and post hoc tests at a confidence level of 95%.

### 4.3. Failure Mode Analysis

After shear bond evaluation, the samples were emersed in methylene blue for 24 h. Later, the teeth were rinsed for 15 minutes under running water and left to dry. Failure modes were observed under a stereomicroscope (Olympus SZ51, Evident, New Delhi, India) at 40× magnification and classified as cohesive within the substrate, adhesive (between cross linker material and dentin), or mixed (if adhesive and cohesive fractures occurred simultaneously).

### 4.4. Specimen Preparation for Scanning Electron Microscopy (SEM) Study 

Five samples from each group were prepared for SEM analysis (Shimadzu SEM EDX, Kyoto, Japan) operated at an acceleration voltage of 15 kV and 10^5^ millibar vacuum pressure. After the occlusal preparation and composite build-up, the samples were sectioned using a double diamond disk with a diameter of 22 mm vertically through the resin build-up and dentin under running water into two halves (mesial and distal) to expose the resin–dentin interface.

Specimens were placed in 4% NaOCl for 20 min, followed by 20% hydrochloric acid for 30 s, and rinsed with distilled water. All samples were then sequentially dehydrated in ascending grades of ethanol, i.e., 60%, 70%, 80%, and 90% alcohol for 20 min each, and 100% alcohol for 1 h. Tested samples were naturally air dried for half an hour and mounted on Aluminium stubs, which were then placed in a vacuum chamber and sputter coated with an Aluminium layer. The bonding interfaces were observed with SEM in order to illustrate the resin/dentin-bonding interface.

### 4.5. Microscopic Evaluation

A series of photographs were taken field by field at a magnification of 2000×, 5000× and 10,000× to view the dentin resin interface.

The length of the resin tags was measured on the photographs with a ruler according to the scale given on the photograph. Scoring criteria were used for visual evaluation, and the four-step (0–3 scale) method according to Ferrari et al. was used for evaluation of the resin-dentin interface [24]. 

### 4.6. Statistical Analysis

The data obtained was entered into an Excel sheet (6.2.8 Excel 2002, v10.0) and analyzed using SPSS version 17 (Chicago, IL, USA). The Mean and SD were calculated, and the data were explored for normality using the Kolmogorov–Smirnov and Shapiro–Wilk tests. A one-way ANOVA was used to find the difference between the groups. A *p* value of ≤0.05 was considered statistically significant.

## 5. Results

### 5.1. Shear Bond Strength 

A one-way ANOVA showed no significant difference in the mean Shear Bond Strength between the different dentine biomodifier experimental groups and the control group (*p* = 0.225). The order of mean Shear Bond Strength in different groups according to the mean values was: GRAPE SEED > CPP-ACP ≅ GLUTERALDEHYDE > HESPERIN > CONTROL (Table 1).

### 5.2. Fracture Mode 

There were three different failure modes in all groups that included adhesive failures (74%), cohesive failures (22%), and mixed failures (4%). 

**Figure 2:**
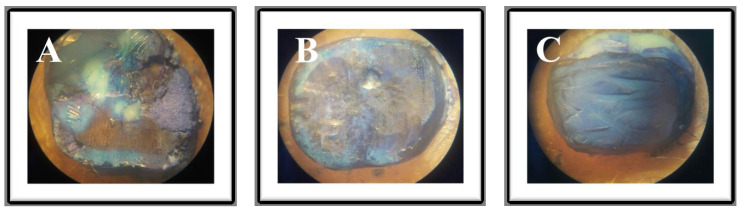
Failure modes (FM) as seen under stereomicroscope at 40× magnification (**A**) Mixed failure (MIX), (**B**) Adhesive failure (ADH), (**C**) Cohesive failure (COH).

Table 2 shows the failure mode of the specimens that were observed under a stereomicroscope. The specimens predominately showed adhesive failure with the highest bond strength values, followed by cohesive failure. Figure 2A–C shows samples of failure modes of the debonded dentin surfaces.

### 5.3. Analysis of the Resin/Dentin Interface

A total of 25 specimens (control and experimental) were taken, and assessment was carried out by two methods: visual inspection and resin tag measurements.

#### Visual Inspection

**Figure 3:**
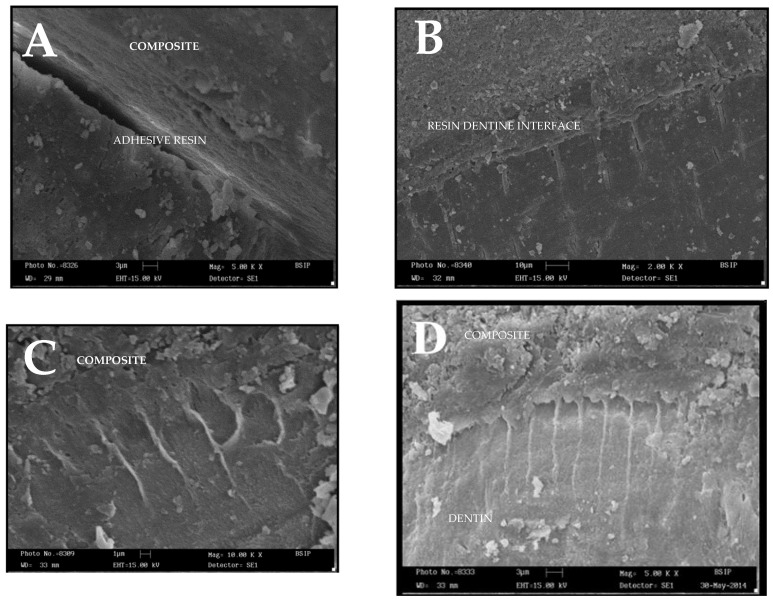
Grading Scores of Different Cross-Linkers as Seen Under Scanning Electron Microscope at a Magnification of 2000×, 5000× and 10,000× (**A**) Grading Score 0, (**B**) Grading Score 1, (**C**) Grading Score 2, and (**D**) Grading Score 3.

Visual inspection of photographs was conducted, and the resin tags in the photographs were graded using a four-step (0–3) scale method proposed. Table 3 and Figure 3A–D show the grading scores obtained for different dentin cross-linkers. As depicted in Table 4, grape seed extract shows the highest grading score of 3. 

### 5.4. Evaluation of Resin Tag Length

Table 4 shows the distribution of the mean length of resin tags in each group, which was statistically significant (*p* < 0.001). Multiple comparisons (Table 5) between the groups using post hoc Least Significant difference (LSD) showed that the mean difference in resin tag length between control and Grape seed (85.20 µm), Control and CPP-ACP (33.60 µm), Control and glutaraldehyde (37.20 µm), and Control and hesperidin (16.20 µm) is significant (*p* < 0.05). The length of resin tags in the experimental groups is significantly greater than in the control group. Thus, from SEM observation, the following order of tag length was as follows: GRAPE SEED > GLUTERALDEHYDE > CPP-ACP > HESPERIDIN > CONTROL.

## 6. Discussion

The present results showed that the incorporation of grape seed extract, glutaraldehyde, hesperidin, and ACP-CPP barely improved the shear bond strength of dentine, as the results were not statistically significant. 

The dentin has 70% of an inorganic phase (hydroxyapatite crystals), 20% of an organic phase of predominantly type I collagen, and 10% water. This assorted composition and hydrophilic nature of dentin limit its consideration as an ideal bonding substrate. 

Enhancing the mechanical properties of Type I collagen may be beneficial during bonding procedures, as it is a major component of the hybrid layer. Dentinal collagen is composed of inter- and intra-molecular cross-links that can increase tensile strength and elasticity. The use of extrinsic collagen cross-linking agents can improve the biomechanical properties of dentin by inducing the additional formation of inter- and intra-molecular cross-links [15]. Very few studies have evaluated the relationship between crosslinking agents and bond strength in primary teeth. Furthermore, the role of bio modifiers as collagen crosslinking agents in primary teeth is little known. Hence, the effect of natural cross-linkers on the shear bond strength and the bonded interface in primary teeth was addressed in the present study.

Ammar et al. (2009) [25] reported that the presence of exogenous cross-links was sufficient to positively affect the mechanical properties of exposed dentine-matrix. 

Natural/ herbal materials have received great attention in the past few years, especially in the field of dentistry. Several synthetic and naturally occurring agents, such as proanthocyanidins, glutaraldehyde, genipin, and carbodiimide, have been used as cross-linkers to protect collagen fibers [14]. The present study observed the effect of cross-linking agents after 1 min of treatment. This procedure could be a leap in pediatric restorative dentistry if adopted and considered a cost-effective method.

Our results showed that the incorporation of grape seed extract, glutaraldehyde, hesperidin, and ACP-CPP barely improved the shear bond strength of dentine, as the results were not statistically significant, and that only the mean values of shear bond strength in comparison to the control group had improved. 

The descending order of mean shear bond strength in different groups according to the mean values was as follows: GRAPE SEED > CPP-ACP ≅ GLUTERALDEHYDE > HESPERIN > CONTROL.

These findings disagreed with previous similar studies investigating the effect of using crosslinking agents to improve both the strength and durability of resin-dentin bonds [16,17,18].

Bedran-Russo et al. (2007) [15] stated that the use of crosslinking agents will increase the strength of the dentin matrix, which in turn might improve the strength of resin-dentin bonds, and they proved that glutaraldehyde was a collagen crosslinker, which increased the stiffness of demineralized dentin. 

Furthermore, dentin biomodification with 6.5% proanthocyanidin was found to have the strongest bond strength in permanent teeth [25,26]. However, when we used the same concentration for treating primary teeth in our study, we did not get the same results. This may be justified as dentin of primary and permanent teeth varies chemically and morphologically in several ways, including lesser mineralization and dentinal tubule concentrations, reduced permeability, higher water content, as well as increased reactivity to acidic conditioners [27]. 

Also considering variation in the symmetry of the width of the peritubular dentin surrounding the tubular lumen in primary teeth when compared to the permanent dentin [28] could be the explanation for ineffectual changes in the bond strength in primary dentition.

In the current study, results showed a statistically significant difference in the mean length of resin tags with different dentine bio-modifiers. The grape seed extract showed very long tags extending almost through the entire resin-dentin inter-diffusion zone, compared to shorter resin tags and lower penetration into the dentinal tubules in the control group. Since most of the retention and sealing effectiveness of dental adhesives is contributed by only the top 5–10 mm of the tags, the actual resin tag length and, in turn, their penetration into the dentinal tubules may not play a significant role in the final bond strength [29]. The major contribution to bonding efficacy is perhaps related to the adaptation to the inner tubule walls.

When types of failure modes were compared, adhesive failure was the most common failure pattern in all groups, corresponding to low bond strength. The SEM analysis revealed that the GD and GSE-treated groups demonstrated a more consistent failure mode. The adhesive layer and the top of the hybrid layer were the most common areas of failure. However, the fractured pattern was observed on the bottom of the hybrid layer in both the control and GE groups. 

### Limitation of the Study

Dentine bond strength does not directly predict clinical performance. Consequently, additional in vivo research is needed to ascertain dentin bond strength and the possible clinical impact of dentine bio modifiers. Since in vitro studies do not replicate real-world settings, long-term in vivo investigations are required. 

Hence, it was concluded that none of the dentine bio modifiers was superior to increasing bonding strength, considering the current data. It is imperative to conduct more studies to increase our understanding of dentin bio modifiers, such as their capacity and concentration to crosslink collagen and strengthen the resin-dentin bond in primary teeth.

## 7. Conclusions

Bio modifiers Incorporation (5% glutaraldehyde, 6.5% grape seed extract, 0.5% hesperidin, and CPP-ACP) in the bonding process for primary teeth did not improve the dentinal bond strength, as no significant difference in shear bond strength was found amongst the control and experimental groups.

## Figures and Tables

**Table 1:** Distribution of Mean ± Standard deviation values of Shear Bond Strength (Mpa) of the control and experimental groups.

	N	Mean ± Std. Deviation	*p* Value
Control	10	120.21 ± 77.65	0.225
Grape seed	10	197.31 ± 12.85	
CPP-ACP	10	150.52 ± 92.61	
Glutaraldehyde	10	150.44 ± 59.52	
Hesperidin	10	122.92 ± 61.97	

**Table 2:** Modes of failure observed in ten samples of the control and experimental groups. Fracture mode (FM), Adhesive failure (ADH), Mixed failure (MIX), Cohesive failure (COH).

Sample No	Control	Grape Seed	Glutaraldehyde	CPP-ACP	Hesperidin
Adhesive failure (ADH)	8	9	7	6	7
Mixed failure (MIX)	1	0	1	0	0
Cohesive failure (COH)	1	1	2	4	3

**Table 3:** Grading scores of the resin tags for control and experimental groups using visual inspection.

Sample No	Control Group (Score)	CPP-ACP Group (Score)	Glutaraldehyde Group (Score)	Grape seed Group (Score)	Hesperidin Group (Score)
1	1	2	2	3	1
2	1	2	2	2	1
3	1	1	2	3	2
4	1	2	1	3	2
5	1	2	2	3	2

**Table 4:** Distribution of Mean values of resin tag length (μm) of the control and experimental groups.

	N	Mean ± Std. Deviation	*p* Value
Control	5	48.20 ± 6.09	<0.001
Grape seed	5	133.40 ± 28.06	
CPPACP	5	81.80 ± 18.29	
Glutaraldehyde	5	85.40 ± 21.22	
Hesperidin	5	64.40 ± 9.55	

**Table 5:** Multiple comparisons of the resin tag length (μm) mean difference between and within the control and experimental groups using post hoc Least Significant Difference (LSD).

Group	Group	Mean Difference	Std. Error	*p* Value
Control	Grape seed	85.20 *	11.66	<0.001 *
	CPPACP	33.60 *	11.66	0.009 *
	Glutaraldehyde	37.20 *	11.66	0.005 *
	Hesperidin	16.20	11.66	0.180
Grape seed	CPPACP	51.60 *	11.66	<0.001 *
	Glutaraldehyde	48.00 *	11.66	0.001 *
	Hesperidin	69.00 *	11.66	<0.001 *
CPPACP	Glutaraldehyde	3.60	11.66	0.761
	Hesperidin	17.40	11.66	0.151
Glutaraldehyde	Hesperidin	21.00	11.66	0.087

* denoted a significant difference at 0.05 level.

## Data Availability

The data presented in this study are available on request from the corresponding author.
